# Nobel-winning microRNA, the micromaestro of gene silencing

**DOI:** 10.1016/j.mocell.2024.100123

**Published:** 2024-10-15

**Authors:** Young-Kook Kim, Jinju Han

**Affiliations:** 1 Department of Biochemistry, Chonnam National University Medical School, Jeollanam-do 58128, Republic of Korea; 2 Graduate School of Medical Science and Engineering, Korea Advanced Institute of Science and Technology (KAIST), Daejeon 34141, Republic of Korea

The Nobel Prize in Physiology or Medicine in 2024 was awarded to Victor Ambros and Gary Ruvkun for their groundbreaking discovery of microRNAs (miRNAs). The miRNAs are a group of small non-coding RNAs that regulate gene expression. The discovery of miRNAs dates back to the early 1990s (Lee et al., 1993, Wightman et al., 1993), but the scientific community required many years to recognize their importance. The discovery by Ambros and Runkun changed our understanding of the regulation of gene expression significantly and revealed an entirely new layer of post-transcriptional regulatory mechanism. Although more than 30 years were required to award their initial finding with the 2024 Nobel Prize, it is noticeable that another RNA-based regulatory mechanism, RNA interference (RNAi), received the same prestigious award earlier (Fire et al., 1998). The back-to-back awarding of 2 closely related fields, thus RNAi in 2006 and miRNAs in 2024, highlights the deep impacts of non-coding RNA-based gene regulatory mechanisms and the long-lasting significance of this field.

The initial works of Ambros’ and Ruvkun’s groups focused on the model organism *Caenorhabditis elegans* (*C elegans*). This fascinating organism, whose cell lineage was fully elucidated during the 1970s and 1980s, serves as an excellent model for studying gene regulation during organ development. In the laboratory of Robert Horvitz, who was awarded the Nobel Prize in 2002 in recognition of his pioneering work using this animal model, Ambros and Ruvkun began their research. They scrutinized *C elegans* mutants, *lin-4* and *lin-14*, which exhibited developmental defects. After obtaining faculty positions, they continued their research and discovered that *lin-4*, a gene involved in developmental timing, did not produce a protein. Unexpectedly, it generated long and short forms of small RNA, initially named small temporal RNA. The short form of this small RNA blocked the translation of lin-14 messenger RNA (mRNA), which contains a complementary sequence to lin-4 in its 3′-untranslated region (Lee et al., 1993, Wightman et al., 1993). This discovery marked the first evidence that small noncoding RNAs could control gene expression at the post-transcriptional level. Although their findings were published in 1993, at that time, their discovery did not get much attention from the broader scientific community. Many researchers thought that this kind of gene regulatory mechanism was unique to roundworms rather than a universal principle.

However, this view was dramatically altered by the discovery of let-7 from Ruvkun’s team in 2000. From their analysis, this miRNA was identified as highly conserved across species, including humans, with conserved target regulation as well. This demonstrates that miRNAs are not unique to roundworms but are indeed essential regulatory molecules in a variety of organisms (Pasquinelli et al., 2000). This discovery revolutionized our understanding of gene regulation, and subsequent discoveries confirmed that miRNAs play a crucial role in diverse biological processes, including development, differentiation, and disease progression. Thus, it became apparent that miRNAs are critical to control complex gene networks across all multicellular organisms.

Interestingly, this year’s Nobel Prize in Physiology or Medicine was awarded nearly 20 years after the Nobel Prize in Physiology or Medicine was awarded to Andrew Fire and Craig Mello for their discovery of RNAi in 2006. RNAi and miRNA are closely related, sharing the protein machinery required for their biogenesis and action. While both reduce the expression of target genes, RNAi induces target mRNA cleavage, whereas miRNAs suppress translation or destabilize target mRNAs (Bartel, 2009). The fact that 2 Nobel Prizes have now been awarded for discoveries in small RNA-based gene regulation mechanisms, thus first for RNAi and then for miRNA, underscores the profound importance of small non-coding RNAs in controlling gene expression, leading to a variety of discoveries that have enriched the field of epigenetics.

Compared to the swift acknowledgment of the discovery of RNAi as the Nobel Prize within a few years after its discovery, it is somewhat surprising that miRNAs, which were discovered earlier, took much longer time to receive the same honor. This delay could partly be attributed to the initial doubts about the universality of miRNAs, as mentioned above. Moreover, RNAi was established rapidly and widely as an essential experimental tool in laboratories soon after its discovery. In contrast, a large number of miRNAs were discovered in roundworms, flies, and humans in 2001 (Lagos-Quintana et al., 2001, Lau et al., 2001, Lee and Ambros, 2001), but their functional studies were relatively slow due to the difficulty in validating the target of miRNAs. Consequently, it took several years to appreciate the broader relevance of miRNAs in human physiology and diseases. With the struggles and efforts of many scientists for years, the importance of miRNAs in organisms has been established well, and the parallels between RNAi and miRNAs highlight the critical roles of RNA in the regulation of gene expression, with both mechanisms now being recognized for their critical impact in biology.

Now, miRNAs are firmly established as key regulators of gene expression, and their significant roles in diverse human diseases have been discovered. Thus, they are extensively studied as biomarkers to diagnose diseases, and many clinical trials target the miRNAs themselves. Although it took a long journey to recognize their importance, miRNA research has deepened our understanding of gene regulation and continues to drive the advance in the development of RNA therapy.

While the contributions of Ambros and Ruvkun are rightly celebrated, it is important to acknowledge that the story of miRNA research is incomplete without acknowledging the pivotal discoveries of key proteins like DROSHA, DICER, and ARGONAUTE, which are indispensable for the biogenesis and function of miRNAs. Both DROSHA and DICER proteins are key ribonucleases in producing the mature miRNAs from their precursor molecules, and ARGONAUTE proteins exert a key role in guiding miRNAs to their target mRNAs resulting in the suppression of gene expression (Kim et al., 2009). Despite their central roles in the miRNA pathway, the discoveries of these proteins have not been acknowledged to the same extent as the initial discovery of miRNAs. Many researchers in the scientific community consider that the critical roles of these proteins also deserve acknowledgment together with the discovery of miRNA.

To sum up, the 2024 Nobel Prize in Physiology or Medicine emphasizes the impact of the discovery by Ambros and Ruvkun on our understanding of RNA-based mechanisms as an essential layer in post-transcriptional gene regulation. This award also prompts a deeper reflection on subsequent discoveries that have expanded our knowledge of miRNA biogenesis and function. As research into miRNAs persists, their roles in both medical diagnostics and treatments are gaining much more attention, with their potential to significantly influence the future of medicine, much like RNAi has done since it was discovered.

## Author Contributions

**Young-Kook Kim:** Conceptualization, Writing – original draft, Writing – review & editing. **Jinju Han:** Writing – review & editing.

## Declaration of Generative AI and AI-assisted technologies in the writing process

During the preparation of this commentary, the authors used ChatGTP 4o to polish the sentences to make it easier for readers to understand the writing. After using this tool, the authors reviewed and edited the content as needed and take full responsibility for the content of the publication.

## Declaration of Competing Interests

The authors declare that they have no known competing financial interests or personal relationships that could have appeared to influence the work reported in this paper.
